# Hypertension secondary to a periprostatic paraganglioma: case report and review of the literature

**DOI:** 10.1186/1472-6823-13-55

**Published:** 2013-11-25

**Authors:** Jesper Kers, Zaheeb A Choudhry, Ton A Roeleveld, Alexander PJ Houdijk

**Affiliations:** 1 Department of Pathology, Academic Medical Center, University of Amsterdam, Meibergdreef 9, 1105, AZ Amsterdam, The Netherlands; 2 Department of Internal Medicine, Medical Center Alkmaar, Alkmaar, The Netherlands; 3 Department of Urology, Medical Center Alkmaar, Alkmaar, The Netherlands; 4 Department of Surgery, Medical Center Alkmaar, Alkmaar, The Netherlands

**Keywords:** Pheochromocytoma, Paraganglioma, Extra-adrenal, Preperitoneal endoscopic resection, Prostate, Lower urinary tract symptoms

## Abstract

**Background:**

Around 10 per cent of catecholamine-secreting tumours can be found outside the adrenal medulla (paraganglioma). We report a case of a functional sporadic paraganglioma that was localized lateral to the prostate without causing lower urinary tract symptoms.

**Case presentation:**

A 76-year old male with an extensive history of cardiovascular disease suffered from hypertension and an unexplained hypochromic microcytic anaemia for years before the coincidental discovery of a 2.5 × 3.5 cm periprostatic mass upon abdominal contrast-enhanced CT scanning. Transrectal biopsies revealed a paraganglioma. The urinary levels of the catecholamine metabolites were found increased. The paraganglioma showed uptake of iodine-123-metaiodobenzylguanidine by SPECT scanning, indicating a solitary lesion. Successful preperitoneal endoscopic resection of the tumour was performed, which resulted in a decrease in blood pressure and a normalization of the urinary catecholamine metabolites. None of the to date known genetic mutations that have been shown to relate to the existence of paragangliomas were identified in the current case.

**Conclusion:**

An intra- or periprostatic localization of a paraganglioma is very rare. We reviewed the literature and found 6 other cases. Three of the described cases presented with lower urinary tract symptoms. In these three patients, the tumour had a size of 4 cm or larger and in 67 per cent of these cases the paragangliomas were situated within the prostate. The periprostatic region might be considered as a possible location for paragangliomas, especially in the presence of lower urinary tract symptoms even though they were absent in the current case.

## Background

Pheochromocytomas and paragangliomas are characterized by a large diversity of clinical features and as a result can remain unrecognized for years. General features of these catecholamine-producing tumours, which originate from the sympathetic nervous system, are related to the response to stress, i.e. paroxysms of hypertension, paleness, headache and sweating attacks [1]. Pheochromocytomas are rare with an incidence of 8 new cases per 100.000 persons per year peaking around the fourth decade of life. The prevalence of pheochromocytoma in patients with hypertension, its major presenting symptom, is around 0.1%. Pheochromocytomas are often found as an incidentaloma of the adrenal medulla upon medical imaging, but in around 10% of cases, they originate from autonomic nervous cells outside the adrenal glands in the neck, the thorax or the abdomen and pelvis (extra-adrenal catecholamine-secreting tunours are referred to as paragangliomas).

In this article, we describe a rare case of a 76-year old male who presented with a sporadic functional paraganglioma that was situated at a very unusual localization, namely lateral to the prostate. A review of the available literature on this specific topic is provided.

## Case presentation

**Figure 1:**
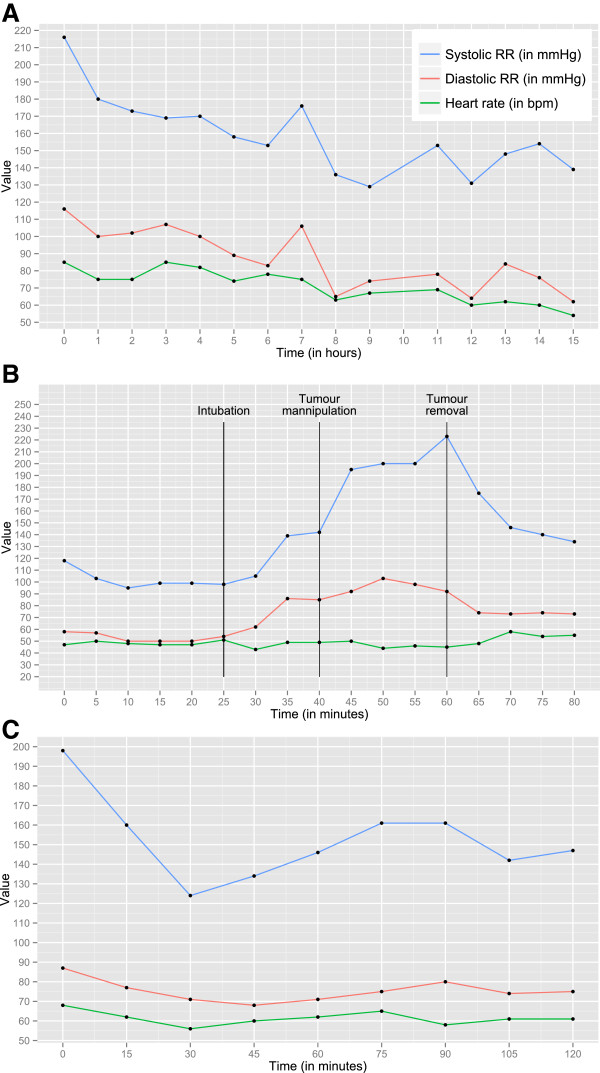
**Blood pressure and heart rate registrations pre-, peri- and post-surgery.** Fifteen-hour registration, starting at 15 h in the afternoon (T = 0) until 6 h in the morning (T = 15), shows a sustained hypertension **(A)**. Blood pressure and heart rate monitoring during the preperitoneal endoscopic resection of the paraganglioma shows an increase in sympathetic activity at start of intubation that is further augmented when the tumour is manipulated **(B)**. When the tumour was removed, sympathetic activity lowered. The heart rate remained constant throughout the procedure. Postoperative monitoring indicates that there is a remainder of systolic blood pressure variability, which is common after removal of paraganglioma **(C)**.

A 76-year old man was referred to the outpatient clinic of the internal medicine department of our hospital because of persisting iron-deficiency anaemia that had been present since 2010. The patient had an extensive history of cardiovascular disease. In 1985 he suffered from an acute myocardial infarction for which he underwent percutaneous transluminal coronary angioplasty of the circumflex branch of the left coronary artery. He was treated for two thromboembolic events: a deep venous thrombosis of his right leg in 2008 and pulmonary embolism in 2009. Over the past years he developed a therapy-resistant hypertension, which was, which was presumed initially due to white coat phenomenon. Figure 1A illustrates the 15-hours continuous blood pressure registration at that time, which did not confirm this hypothesis, showing a sustained hypertension pattern. In 2010 he was diagnosed with hypertensive cardiomyopathy that resulted in decompensated heart failure two years later. Furthermore, the patient had a history of type 2 diabetes mellitus, hypercholesterolemia, psoriasis and macular degeneration. His anti-hypertensive medication consisted of amlodipine/valsartan/hydrochlorothiazide 1 dd 5/160/12.5 mg, furosemide 1 dd 40 mg and nebivolol 1 dd 5 mg. Furthermore he was using atorvastatine 1 dd 40 mg, carbasalate calcium 1 dd 100 mg, metformine 2 dd 500 mg, paroxetine 1 dd 10 mg, oxazepam 1 dd 10 mg, pantoprazole 1 dd 40 mg, ferrous fumarate and rizatriptan 10 mg whenever needed.

**Figure 2:**
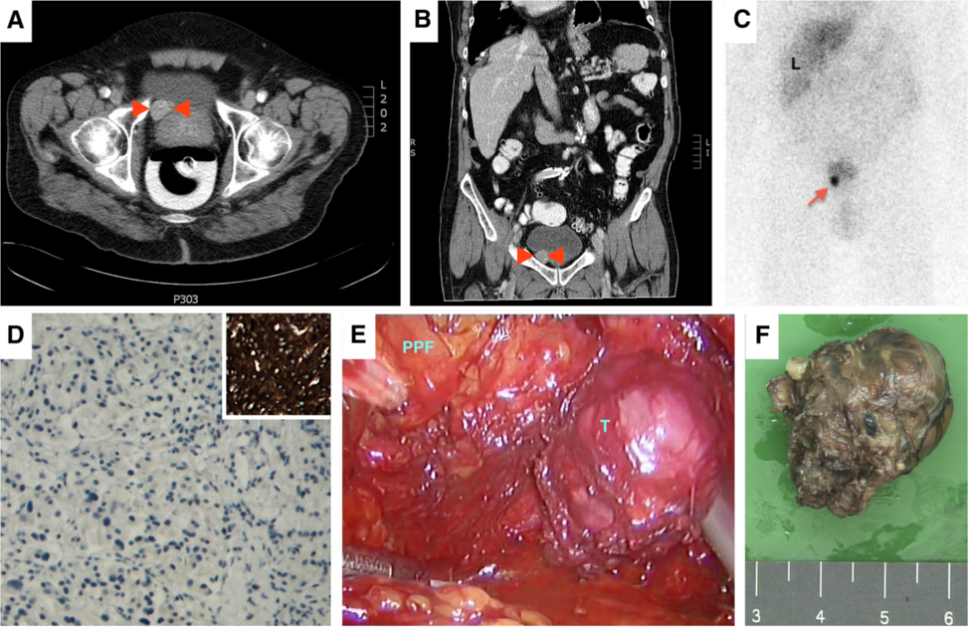
**Abdominal computed tomography, **^**123**^**I-MIBG scintigraphy, pathology and preperitoneal endoscopic resection of the pheochromocytoma.** Axial **(A)** and coronal view **(B)** on abdominal computed tomography with intravenous and rectal contrast shows a round-shaped, sharply demarcated tumour of 1.7 by 2.5 cm possibly attached to the bladder wall just lateral to the prostate. No local (or distant) lymphadenopathy was observed. Arrowheads in red indicate the tumour. The iodine-123-metaiodobenzylguanidine (^123^I-MIBG) SPECT-scan shows high uptake of ^123^I in the periprostatic tumour without suspicion of catecholamine-producing tumours located elsewhere in the body **(C)**. Furthermore, aspecific uptake can be observed in the liver and also the bladder through renal clearance of the (coupled) isotope. “R” indicates the right side of the patient and “L” indicates the liver. The red arrow indicates the tumour uptake of ^123^I-MIBG. Hematoxylin staining of the transrectal biopsies of the tumour showed epithelial cells with dark nuclei that differed in size with local tube formation **(D)**. In the cytoplasm of the epithelial cells, a granular pattern was observed. Immunohistochemistry of the tissue shows a positive staining for chromogranin A, CD56 and synaptofysin. Staining for PSA and PSAP, which might be indicative of prostatic tissue, were negative. The inlet illustrates the chromogranin A immunostaining on the tumour tissue. Snapshot of the camera during preperitoneal endoscopic resection of the paraganglioma shows the close proximity to the prostate **(E)**. The peri-prostatic fat is indicated in light-blue by “PPF” and the tumour by “T”. Gross macroscopy of the 3.5 by 2.5 by 2 cm partly encapsulated tumour that was resected via preperitoneal endoscopic surgery **(F)**.

Upon arrival at the medical outpatient clinic, he complained of dyspnea on exertion that had been stable since he was diagnosed with decompensated heart failure. He also complained of paroxysmal headaches without aberrant palpitations, increased transpiration or paleness. In the context of the persisting anaemia, which was the initial reason for referral, he had not noticed a change in his defecation pattern over the past years and especially no melaena or bloody stool had been present. Further laboratory investigation showed a low mean corpuscular volume and hemoglobin concentration with a low serum iron and ferritin, but a high iron-binding capacity, indicative of hypochromic microcytic anaemia in the context of an iron deficiency (Table 1). The absolute erythrocyte count was within the normal range. In 2010, no cause for his anaemia was found on gastroduodenoscopic and colonoscopic evaluation. A screening CT-scan of the thorax and abdomen with intravenous and rectal contrast was performed to screen for any possible primary malignancy. The abdominal CT-scan showed a round and sharply demarcated tumour of 1.7 by 2.5 cm in close proximity to the bladder wall on the right side of the patient’s prostate (Figure 2A and B). The average post-contrast radiodensity of the tumour measured 90 Hounsfield units, which did not have additional value for the differential diagnosis due to the contrast. No local lymphadenopathy was detected. The findings of the CT-scan were confirmed by digital rectal examination, where an asymmetrically and irregularly enlarged tumour with a hard node was felt and the patient was referred to the urologist for further evaluation under the suspicion of a prostate tumour.

**Table 1:** Chemical, haematological and endocrinological work-up on initial presentation at the medical outpatient clinic

**Serum chemistry**	**Value**	**Reference**
Sodium (mmol/L)	135	135 – 145
Potassium (mmol/L)	4.5	3.5 – 4.5
Calcium (mmol/L)	2.66 (corr. 2.68) ⬆	2.10 – 2.55
Phosphate (mmol/L)	0.8 ⬇	0.9 – 1.5
Magnesium (mmol/L)	0.9	0.7 – 1.0
Albumin (g/L)	39	35 – 55
Creatinine (μmol/L)	88	80 – 125
eGFR CKD-EPI (mL/min/1.73 m^2^)	73	>60
Morning glucose	5.9	4.0 – 6.4
HbA1c (mmol/mol)	41	<53
Iron (μmol/L)	5 ⬇	14 - 35
Total iron-binding capacity (μmol/L)	75.3 ⬆	27 – 54
Ferritin (μg/L)	16 ⬇	25 – 250
Folic acid (nmol/L)	55.7 ⬆	5 – 23
Vitamin B_12_ (pmol/L)	364	130 – 700
Alkaline aminotransferase (U/L)	18	<50
Aspartate aminotransferase (U/L)	12	<45
Alkaline phosphatase (U/L)	89	<125
Gamma-glutamyltransferase (U/L)	67 ⬆	<45
Total bilirubin (μmol/L)	7	<17
C-reactive protein (mg/L)	4.6	<10
25-hydroxy vitamin D (nmol/L)	19 ⬇	20 – 100
**Hematology**		
Hemoglobin (mmol/L)	7.4 ⬇	8.5 – 11.0
Mean corpuscular volume (fL)	79.9 ⬇	82 – 98
Mean corpuscular hemoglobin (fmol)	1.6 ⬇	1.7 – 2.1
Erythrocyte count (10^12^/L)	4.73	4.3 – 6.0
Erythrocyte sediment rate (mm/uur)	53 ⬆	<20
Thrombocytes count (10^9^/L)	381	150 – 400
Leukocyte count (10^9^/L)	7.6	4.0 – 10.0
**Endocrinology**		
Thyroid-stimulating hormone (mU/L)	2.3	0.5 – 3.9
Free thyroxine (pmol/L)	10.5	9 – 24
Parathyroid hormone (pmol/L)	9.4 ⬆	2 – 7
Calcitonin (pmol/L)	5.1	<25
Prostate-specific hormone (μg/L)	2.5	<6.5
**Urine (24-hours collection)**		
Calcium (mmol/L)	4.3	3.5 – 8.0

When he presented at the urology department, his serum total prostate-specific antigen concentration was 2.5 μg/L (reference value for the age of 70 – 80 < 6.5 μg/L). At that time, the patient had no lower urinary tract symptoms and his headache had not worsened during micturition. Cystoscopy revealed an impression of the right bladder wall with no mucosal pathology. Three transrectal ultrasonography-guided biopsies of the lesion where obtained and histological analysis of the material with hematoxylin staining showed partly tube-forming tumorous epithelial cells with intensely stained nuclei that differed in size (Figure 2D). Immunohistochemical analysis of the tissue showed hardly any proliferating cells by Ki-67 staining, but intense staining for chromogranin A (Figure 2D, inlet), synaptofysin and neural cell adhesion molecule (NCAM), consistent with the phenotype of a paraganglioma. Immunohistochemistry for markers of prostatic tissue to rule out prostatic cancer, i.e. prostate-specific antigen (PSA) and prostate-specific acid phosphatase (PSAP), were both negative. Further laboratory investigation to determine catecholamine excess was performed by 24-hours urine analysis on their metabolites. In two separate 24-hours urine samples, the concentrations of the fractionated metanephrines were found increased (Table 2), which could not be explained by a concomitant psychiatric illness the use of sympathomimetic drugs or medicaments. Monoamine oxidase inhibitors were not used. SPECT-scanning with iodine-123-metaiodobenzylguanidine (^123^I-MIBG), a molecule similar to norepinephrine and taken up in adrenergic tissue showed pathological uptake solely within the paraganglioma (Figure 2C). By evaluation of the patient’s serum, hyperparathyroidism was found that could be, in combination with the paraganglioma, indicative of a multiple endocrine neoplasia 2a syndrome (MEN2a). Additional serum analysis showed a corrected calcium concentration of 2.68 mmol/L and moderate hypovitaminosis D (Table 1). A sestamibi parathyroid scintigraphy was performed with ^99m^Tc-MIBI and substraction with ^123^I that showed no washout suspected for parathyroid adenoma or hyperplasia. Under the provisional diagnosis of secondary hyperparathyroidism, vitamin D was supplemented, which resulted in a normalization of the corrected serum calcium concentration to 2.45 mmol/L. With a serum calcitonin concentration of 7.4 pmol/L (reference <24 pmol/L) and a carcinoembryonic antigen (CEA) concentration of 3.3 μg/L (reference in non-smokers < 5.0 μg/L), medullary thyroid cancer became less likely. Since extra-adrenal pheochromocytomas have been shown to be more prevalent in patients with a hereditary paraganglioma syndrome, genetic screening was indicated. Various schemes for genetic screening based on risk factors for mutations have been proposed, which include screening for *RET*, *SDHB*, *SDHD* and *VHL* in case of abdominal paragangliomas [2,3]. Erroneously, all to date known genes that are related to pheochromocytomas and paragangliomas were sequenced, including multiplex ligation-dependent probe amplification (MLPA) to detect larger deletions (*RET*, *MAX*, *SDHA*, *SDHAF2*, *SDHB*, *SDHC*, *SDHD*, *TMEM127* and *VHL;* MRC-Holland kit P226-B2), but none showed pathogenic mutations in their coding sequence or splice sites.

**Table 2:** Urinalysis of metabolites of the catecholamines

	**Pre-operative**		**Post-operative**		
	**First urinalysis**	**Second urinalysis**	**2 weeks urinalysis**	**5 months urinalysis**	**Reference**
Metanephrine (μmol/24 h)	1.1	2.4	0.9	1.2	<2.0
Normetanephrine (μmol/24 h)	7.1	16.8	5.7	4.9	<5.0

The patiënt was scheduled for preperitoneal endoscopic resection of the paraganglioma and Figure 2E shows a snapshot of the peri-prostatically localized tumour via the endoscopy camera. Prior to surgery the patient’s blood pressure was lowered according to the scheme proposed by Pacak [4]. First, 4 weeks prior to surgery nebivolol was halted due to the chance of paradoxical hypertensive crises with beta blockade. Alpha blockade with doxazosine was initiated and increased up till 1 dd 32 mg. Secondly, beta-blockade with metoprolol retard 1 dd 50 mg and subsequently nifedipine retard 1 dd 30 mg were added to the regimen, which resulted in a pre-operative blood pressure of 140/80 mmHg. Pre-operative resuscitation with NaCl 0.9% was performed in order to reduce intravascular dehydration. Blood pressure was closely monitored pre-, per- and post-operation. At the start of intubation by the anesthesiologist, blood pressure started to rise (Figure 1B). Preperitoneal carbondioxide inflation caused the blood pressure to increase by another 30% systolically and diastolically and manipulation of the paraganglioma resulted in a systolic and diastolic blood pressure above 230 and 100 mmHg, respectively. After removal of the paraganglioma systolic and diastolic blood pressure dropped (Figure 1B). During 2 hours post-operative monitoring, blood pressure remained between 120 and 160 mmHg systolically and 60 and 90 mmHg diastolically (Figure 1C). Two days after surgery, his blood pressure could be adequately regulated by metoprolol only. One month after surgery, the average blood pressure under metoprolol treatment was 160/100 mmHg with a heart rate of 90 beats per minute and hydrochlorothiazide/valsartan 1 dd 12.5/80 mg was initiated, which resulted in blood pressure of 150/90 mmHg. Pathologic examination of the excised tumour, which had a diameter of 2.5 - 3.5 cm on gross macroscopy, confirmed the diagnosis of paraganglioma (Figure 2F). One year after resection of the paraganglioma, the patient was readmitted to the medical ward again with a microcytic anaemia and reticulocytosis. Under the suspicion of gastrointestinal blood loss, gastroduodenoscopy and colonoscopy plus videocapsule endoscopy have been planned. To date, the microcytic anaemia is not believed to be associated with the paraganglioma.

## Conclusions

Here we documented the case of a 76-year old male with symptoms of sustained therapy-resistant hypertension, unexplained microcytic anaemia and a catecholamine-producing tumour near the prostate that had remained unrecognized for years. The paraganglioma was discovered incidentally on a screening abdominal CT scanning made during the work-up for his unexplained and presumably unrelated anaemia.

To the best of our knowledge, this is the seventh case in history reporting a paraganglioma that is localized in or in close proximity to the prostate [5-10]. Compared to the other described cases, the current patient is relatively old at time of diagnosis and the tumour size was the smallest in the range (Table 3). None of the cases mentioned (microcytic) anaemia at time of diagnosis. Remarkably, three of the described cases presented with symptoms related to micturition; in two of these cases the paragangliomas were situated within the prostate and all cases that had complaints related to micturition had a tumour size of 4 cm or larger (Table 3). Of the 7 cases, 5 patients (71%) originated from Europe.

**Table 3:** Comparison of cases of (peri)prostatically localized pheochromocytomas from the literature

**Citation**	**Country of origin**	**Age**	**Family history of pheochromocytoma**	**Presenting symptoms**	**Related to micturation**	**Multi-focal**	**Tumour location**	**Maximum tumour diameter**
Pichat *et al*. [5]	France	15 yr	N.D.	Sustained hypertension, hyperglycemia and headache after micturition	Yes	No	Intraprostatic	4 cm
Nielsen *et al*. [6]	Denmark	37 yr	N.D.	Sustained hypertension, sweating and paroxysmal headaches	No	Yes	Intraprostatic	3.5 cm
Dennis *et al*. [7]	United States of America	35 yr	Yes	Paroxysmal hypertension	Yes	No	Intraprostatic	5 cm
Voges *et al*. [8]	Germany	8 yr	No	Paroxysmal hypertension, headaches, and blurred vision	No	Yes	Intraprostatic	N.D.
Perlmutter *et al*. [9]	United States of America	63 yr	N.D.	Sustained hypertension, exacerbated by urination	N.D.	No	Lateral (left), periprostatic	3.9 cm
Padevit *et al*. [10]	Switzerland	41 yr	No	Paroxysmal headaches, dizziness, nausea and syncope during micturation	Yes	No	Anterolateral (right), periprostatic	6 cm
Kers *et al*. [11]	The Netherlands	76 yr	No	Paroxysmal headaches, hypertension and iron-deficiency anemia	No	No	Anterolateral (right), periprostatic	3.5 cm

N.D. = not described.

Besides the classical triad of paroxysmal palpitations, headache and sweating, which are direct signs of sympathetic activation [1], pheochromocytomas and paragangliomas may present with a variety of cardiovascular manifestations related to this classical triad as well [12,13]. Interestingly, our patient did not present with any of the classical symptoms related to catecholamine excess and is primary symptom was a sustained therapy resistant hypertension. In a report by Zelinka and colleagues, 19% of patients that later presented with a functional pheochromocytoma had prior cardiovascular complications that included arrhythmias, tako-tsubo-like cardiomyopathy and even myocardial infarction and stroke [13]. In the case presented in the current manuscript, the patient had been diagnosed with hypertensive cardiomyopathy two years before the diagnosis of paraganglioma, which is in line with the report by Zelinka and colleagues.

Extra-adrenal localization of catecholamine-secreting tumours is more often in the context of a genetic syndrome and it is therefore recommended to screen these patients for underlying genetic mutations [2]. The current report is the first in the literature that describes screening in a case of a (peri)prostatically localized paraganglioma [1,14]. By mistake, the full spectrum of to date known germline mutations was analysed, which is not cost effective. In the current patient, the internally validated scheme by Erlic et al. advices to start with screening for SDHB followed by VHL, SDHD and RET in case of a single paraganglioma, which is not located in the head and neck region [2]. We advice to follow risk factor guided mutation screening, since these schemes were shown to have a high c-statistic for detection of germline mutations and costs can be reduced up till 40% as compared to a screening in predefined order (SDHB > VHL > RET > SDHD) in each patient presenting with a catecholamine-secreting tumour.

In conclusion, we describe the case of a functional periprostatic pheochromocytoma, which is an unusual extra-adrenal site of presentation for this type of neoplasia. The tumour was recognized on a contrast-enhanced CT scan performed in the work up for unexplained and presumably unrelated microcytic anaemia. Although very rare, the periprostatic region should be considered as a possible location for paragangliomas, especially in the presence of lower urinary tract symptoms even though they were absent in the current case.

### Consent

Written informed consent was obtained from the patient for publication of this case report and any accompanying images.

## Abbreviations

NCAM: Neural cell adhesion molecule; PSA: Prostate-specific antigen; PSAP: Prostate-specificacid phosphatase; VMA: Vanillyl mandelic acid; MEN: Multiple endocrine neoplasia; SPECT: Single photon emission computed tomography; MIBG: Iodine-123-metaiodobenzylguanidine; PRRT: Peptide receptor radionuclide therapy; MIBI: Methoxyisobutylisonitrile; CEA: Carcinoembryonic antigen; RET: Ret proto-oncogene; MAX: Myc-associated factor X; SDH: Succinate dehydrogenase; TMEM127: Transmembrane protein 127; VHL: von Hippel-Lindau.

## Competing interests

None of the authors have competing interests to declare.

## Authors’ contributions

JK: contributed to study conception and design, acquisition of the data, analysis of the data, drafting and critically revising of the manuscript and has given final approval of the version to be published. ZAC: contributed to acquisition of the data, drafting and critically revising of the manuscript and has given final approval of the version to be published. TAR: contributed to acquisition of the data, drafting and critically revising of the manuscript and has given final approval of the version to be published. APJH: contributed to acquisition of the data, drafting and critically revising of the manuscript and has given final approval of the version to be published. All authors read and approved the final manuscript.

## Disclosure statement

The authors have nothing to disclose.

## Pre-publication history

The pre-publication history for this paper can be accessed here:

http://www.biomedcentral.com/1472-6823/13/55/prepub
